# The Concentration of Selected Heavy Metals in Muscles, Liver and Kidneys of Pigs Fed Standard Diets and Diets Containing 60% of New Rye Varieties

**DOI:** 10.3390/ani11051377

**Published:** 2021-05-12

**Authors:** Agnieszka Chałabis-Mazurek, Jose Luis Valverde Piedra, Siemowit Muszyński, Ewa Tomaszewska, Sylwia Szymańczyk, Sylwester Kowalik, Marcin B. Arciszewski, Anna Zacharko-Siembida, Tomasz Schwarz

**Affiliations:** 1 Department of Pharmacology, Toxicology and Environmental Protection, University of Life Sciences, Lublin, 13 Akademicka St., 20-950 Lublin, Poland; agnieszka.mazurek@up.lublin.pl; 2 Department of Biophysics, Faculty of Environmental Biology, University of Life Sciences in Lublin, 13 Akademicka St., 20-950 Lublin, Poland; siemowit.muszynski@up.lublin.pl; 3 Department of Animal Physiology, Faculty of Veterinary Medicine, University of Life Sciences in Lublin, 12 Akademicka St., 20-950 Lublin, Poland; ewaRST@interia.pl (E.T.); sylwia.szymanczyk@up.lublin.pl (S.S.); sylwester.kowalik@up.lublin.pl (S.K.); 4 Department of Animal Anatomy and Histology, University of Life Sciences in Lublin, 20-950 Lublin, Poland; mb.arciszewski@wp.pl (M.B.A.); asiembida13@wp.pl (A.Z.-S.); 5 Department of Animal Genetics, Breeding and Ethology, Faculty of Animal Sciences, University of Agriculture in Kraków, 24/28 Mickiewicza Ave., 30-059 Cracow, Poland; rzschwar@cyf-kr.edu.pl

**Keywords:** heavy metals, toxicology, pigs nutrition, rye, hybrid rye

## Abstract

**Simple Summary:**

Modern varieties of cereal grains commonly cultivated in Europe are considered good alternative energy sources that can be used as partial replacements for barley and wheat, which are commonly used as primary energy sources in pig feed. In Central Europe, rye deserves special consideration as it can be cultivated on low-fertility soils with a very low environmental impact among cereals. The objective of the present study was to evaluate the effect of partial substitution of wheat and barley with maize or modern rye varieties (population and hybrid) on concentration of selected heavy metals in feed offered to pigs and in pig muscles, liver and kidneys at slaughter.

**Abstract:**

The carry-over of heavy metals from feed to muscles is generally low if animals are fed with a standard diet containing amounts below the maximum permissible levels. However, prolonged exposure to heavy metals can lead to their accumulation in some organs like muscles, liver, and kidneys. This paves the way for human health risks related to the consumption of products of animal-origin. Thus, using feed mixtures with a low level of heavy metals in pig production will contribute to increasing public health and safety and is of environmental concern. The study aimed to assess the impact of the level of some heavy metals (Cd, Pb, Hg, Cu, Zn, Fe and Mn) in standard (control) feed mixtures and in alternative feed mixtures based on maize or new rye varieties (population and hybrid) on the heavy metal concentration in muscles, liver and kidney of fattened pigs at slaughter. While some differences between heavy metals content in examined tissue samples from experimental groups were observed, all of them were in the range of allowable levels according to European Community rules. In conclusion, new rye varieties, especially the hybrid variety, could be an alternative source of cereal grains for pig nutrition.

## 1. Introduction

Among the other cereal species, rye is less popular worldwide than maize or wheat, however it is substantially important locally in countries with specific soil and climate conditions, especially in Central and Eastern Europe, the regions forming so-called Rye Belt, and Canada [1]. Three largest producers of rye presently are Germany, Poland and Russia [2]. Typically, rye cultivars are considered as belonging to one of two types: population rye or hybrid rye, which are different in some characteristics [3]. However, in many countries the third type is also popular in cultivation, no name rye (NN rye), meant as rye grown from the seeds which do not qualify as a registered variety. Such seeds, after many years of farm own multiplication have no stabile properties in case of yielding efficiency and nutritional value. The rate of the usage of unqualified seeds is the greater the farther to East, thus the lowest in Germany, intermediate in Poland, and the largest in Russia, Belarus and Ukraine [2]. Rye is the most resistant cereal for poor sandy soils, freeze and deficiency of water (having the lowest transpiration coefficient, rye use relatively low amount of water) thus every specific condition for the aforementioned European countries. Rye is also the most resistant for pests and molds, excluding only ergot [3,4]. That is why rye is cultivated on poorest soils, using the lowest value of agricultural chemistry, like artificial fertilizers, pesticides and fungicides. The direct effect of those properties is the economic advantage of rye cultivation and usage [5,6,7,8]. Indirectly, it seems probable that the value of rye seed pollution with heavy metals and pesticides wastes could be lower than in others, more demanding cereal species.

Toxic heavy metals like cadmium (Cd), lead (Pb), and mercury (Hg) are naturally present in the Earth’s crust at varied quantity. The anthropogenic activities like mining and production of fertilizers and pesticides contribute to the increasing level of environmental pollution with these metals. Cereals may contain the largest quantities of Cd and Pb since they are rapidly absorbed by plants from soil. The bioavailability of heavy metals for the crop plants depends on soil physicochemical features and plants predilection to their increased uptake and organs accumulation and that facilitate their entrance into the food chain [9]. In animals, these heavy metals are not required for the physiological function. Thus, they are undesired elements in animal feed and are classified as contaminants [10].

Heavy metals bind to structural proteins, enzymes, and nucleic acids, and interfere with the functioning of cells. Due to the long biological half-life of heavy metals and their ability to accumulate in animal and human bodies, they may cause chronic toxicity [11]. Symptoms and effects can vary according to the metal or metal compound, and the dose involved. Broadly, long-term exposure to toxic heavy metals can have carcinogenic, central and peripheral nervous system and circulatory effects [12]. Cadmium shows genotoxic, mutagenic, teratogenic effects in humans and animals [13]. Additionally, its accumulation impairs the process of vitamin D metabolism and, consequently, reduces the absorption of calcium from the intestinal tract. Lead after absorption from the gastrointestinal tract, it is transported first to the liver, where it accumulates, next to the kidney, heart, and brain, and later to the muscle or bone tissue. Long-term Pb and Cd intake can also alter the organization of intestinal mucosa [14]. The mechanism of toxicity of these heavy metals is also linked to bone damage through the substitute of divalent calcium due to structural similarities [15].

By contrast, some heavy metals are required in small quantities (i.e., micronutrients) for both human and animal health. These elements, essential trace elements, include among others, manganese (Mn), iron (Fe), copper (Cu), and zinc (Zn) [16,17]. A deficiency of these essential metals may increase susceptibility to heavy metal poisoning [18,19,20]. According to the European law [21] essential heavy metals are allowed to be used in animal nutrition to optimize animal production.

Under physiological conditions plasma Cu levels oscillate from 100 to 130 μg/100 mL. Cu plays the role as an enzyme activator participating in reduction–oxidation processes. Copper and iron take part in initiating the generation of reactive oxygen species (ROS), which react with polyunsaturated fatty acid residues of cell membranes, thiol-containing proteins, and nucleic acids leading to oxidative stress and cytotoxic effects. On the other hand, copper is required for the activity of superoxide dismutase (SOD), which is a scavenger of reactive oxygen metabolites, which can be induced by undesired heavy metals. Monogastric animals given digestible diets, absorb about 60% of copper intakes. Common diets for fattening pigs, based on grain, are quite rich in this mineral, since the protein sources usually contain enough copper [20].

Manganese (Mn) as an essential trace element is present in all tissues but especially highly concentrated in bones, liver and kidneys. About 3% of ingested Mn is absorbed and distributed to different compartments of the body and its turnover rate depends on the level of dietary intake. Manganese is essential for the proper development and growth of the skeletal system. Manganese poisoning requires the intake of large amounts of this element. Excessive Mn intake causes elevated concentrations of the metal in the liver, alteration of copper distribution, drastic reduction in iron absorption and reduced calcium and phosphorus excretion [20].

The major content of zinc (Zn) in the body is found in the muscles and bones. It is a cofactor of many enzymes, among which the most common are carbonic anhydrase, carboxypeptidase, alkaline phosphatase, alcohol dehydrogenase, glutamine dehydrogenase, lactate dehydrogenase and RNA polymerase. Zn optimal tissue concentration determines the precise structure of skin and mucosa and guarantees physiological development and growth of the skeletal system and the whole organism [20]. Different studies reported that the carry-over of heavy metals to muscles is generally low if animals are fed with a standard diet containing amounts below the maximum permissible levels. However, prolonged exposure to heavy metals can lead to their accumulation in some organs like muscles, liver, and kidneys [9,22]. It paves the way for human health risks related to the consumption of products of animal origin. Therefore, using feed mixtures with low level of heavy metals in pig production will contribute to increase public health and safety and is of environmental concern.

While the effects of feed ingredients on performance and meat chemical composition are commonly evaluated [23,24,25], the concentration of heavy metal in muscles or internal organs is less extensively studied. Moreover, the inclusion of rye grain at the level of 60% in the pig feed mixtures has not been studied before. The analyses of toxic heavy metals concentration will enable to define the level of pollution transfer from animal feed to organism of pigs, what is important for human safety. Therefore, the aim of the study was to assess the effect of 60% inclusion of new rye varieties in feed mixtures on the heavy metal concentration both toxic (Pb, Cd, Hg) and essential (Cu, Zn, Fe, Mn) in chosen organs (muscles, liver, and kidney) of fattened pigs at slaughter.

## 2. Materials and Methods

### 2.1. Animals and Experimental Design

The study was performed at the National Research Institute of Animal Production’s Experimental Station in Chorzelów (Poland). In total, 100 Polish Landrace pigs of both sexes (50 barrows and 50 gilts) weighing 30 ± 1.0 kg were used in this study. All animals were ear marked by tattooing. Pigs were randomly divided into four groups (one control and three experimental groups, *n* = 25 of each group) and housed in controlled fattening individual balance cages.

The diet for animals from the experimental groups contained 60% of maize (as control diet for 60% grain inclusion), 60% of population rye (cv. Dankowskie Granat) and 60% of hybrid rye (cv. Binntto), respectively (Table 1 and Table 2). All diets were formulated to meet or exceed national requirement with regards to nutrients, metabolizable energy and mineral elements for pigs [26]. All cereal components of control and experimental diet were purchased from KWS Lochów Polska Sp. z o.o. (www.kws-zboza.pl, accessed on 31 March 2021) and contained a multienzyme Enzyme G2G (betaglucanase and xylanase, 20,000.0 mg/kg, BAS-POL, Zębowice, Poland) preparation for pig’s rations. For determination of heavy metal content in cereals and feed, samples of cereal grains and feed mixtures from three random location within each batch were collected and individually packed into plastic bags.

All animals were fed with the corresponding diet (grower for 40 days and finisher for 32 days) until they reached final body weight of approximately 100 kg. At the end of the experiment the animals were weighed and killed in a local slaughterhouse. Samples of diaphragmatic muscles, liver and kidneys were collected during routine veterinary inspection individually from eight representative pigs, with the body weight closest to the group average, in each group. A set of total 96 tissue samples (*n* = 3 tissue fragments from *n* = 8 pigs from each of *n* = 4 experimental group) was obtained. Each sample was individually packed into labeled plastic bags and transferred to laboratory for further analysis.

### 2.2. Samples Preparation and Analysis

From each collected tissue samples, a minimum 5 g representative sample was taken and deeply frozen, freeze-dried and homogenized. Similarly, the samples of cereal grains and feed mixtures were homogenized by vibrating ball mill to obtain homogenous material. Dry mineralization process of samples was performed prior to elements analysis. For this purpose, 1.0 g sub-samples of cereal grains and feed mixtures, and 0.5 g of samples of dry tissues were weighed with accuracy of ±0.0001 g and mineralized in an electric stove using final the temperature of 450 °C. The ash obtained was dissolved with a mixture of concentrated nitric acid (65%) of spectral purity (Merck, Darmstadt, Germany) with re-distilled water in proportion 1:1 for further analysis of elements.

The levels of Zn and Fe were determined using the method of flame atomic absorption spectrometry (Avanta PM, GBC, Melbourne, Australia), while Pb, Cd, Cu and Mn contents were assayed by the use of an atomic absorption spectrometer (GFAAS) with electrothermal atomization and Zeeman background correction (SpektrAA 220Z, Varian, Palo Alto, CA, USA). A palladium solution (Merck, Darmstadt, Germany) was used as a chemical modifier for the analysis of Cd, and NH_4_H_2_PO_4_ (Merck, Darmstadt, Germany) for Pb.

Mercury level in samples was measured by cold-vapour atomic absorption spectrometry with the MA-2000 system (Nippon Instruments Corporation, Takatsuki, Japan) at 253.7 nm, where mercury was determined without sample pre-treatment. The homogenized samples were directly weighed (10–100 ± 0.1 mg) into pre-cleaned combustion boats and inserted into automatic mercury analyzer.

The methods were controlled by analyzing the series of samples from a two certified reference material (Pig Kidney CRM 186 and Rye Grass ERM 281, Institute for Reference Materials and Measurements, European Commission, Geel, Belgium). Recoveries between 90 and 110% were accepted to validate the calibration for all elements. The limits of quantification (LOQ) were as follows: 0.027, 0.201, 0.024 and 0.027 mg/kg for Zn, Fe, Cu and Mn, respectively. The LOQ for Pb and Cd were 0.003 and 0.0003 mg/kg, respectively, while for Hg 0.0003 mg/kg.

The analyses of the content of analyzed elements in the raw materials used for the preparation of the feed mixtures are presented in Table 3, while the content of heavy metals in the grower and finisher feed rations are shown in Table 4 and Table 5, respectively. The level of mercury was below LOQ in all grain and feed samples.

### 2.3. Statistical Analysis

Data are expressed as lsmeans and SEM (standard error of means) with *n* = 8 in each group. All statistical procedures were conducted using Statistica 13.3 software (TIBCO Software Inc., Palo Alto, CA, USA). Normal distribution of data was examined using the Shapiro–Wilk test and equality of variance was tested by the Levene’s test. For normally distributed data, a one-way analysis of variance (ANOVA) with Tukey’s honestly significant difference (HSD) post hoc test was used. For data that did not meet the assumptions for parametric tests, a non-parametric Kruskal–Wallis ANOVA with Dunn’s post hoc test was used. The relationships between the assessed heavy metals content of the tested tissues were also estimated by means of Spearman’s rank correlation coefficients. For all tests, a *p*-value of less than 0.05 was considered statistically significant.

## 3. Results

### 3.1. Concentration of Heavy Metals in Muscles

The concentration of heavy metals in the diaphragmatic muscles of control and experimental pigs (Table 6) did not exceed the permissible levels in the European Union (EU) [21]. However, the highest concentration of cadmium was found in the muscles of pigs fed the population rye mixture (0.021 mg/kg), which was significantly higher than that found in the muscles of pigs feed the hybrid rye mixture (0.008 mg/kg, *p* < 0.001), maize mixture (0.006 mg/kg, *p* < 0.001), and the control mixture (0.003 mg/kg) (Table 6). Lead concentration in the diaphragmatic muscles was significantly higher in all pigs fed experimental mixtures: maize mixture (0.37 mg/kg, *p* < 0.01), population rye mixture (0.27 mg/kg, *p* < 0.01), and hybrid rye mixture (0.42 mg/kg, *p* < 0.01) compared to pigs fed the control diet (0.09 mg/kg). The concentration of mercury was the highest in diaphragmatic muscles of pigs fed the maize mixture (0.032 mg/kg) and it was significantly different from the concentration in pigs fed population rye mixture (0.004 mg/kg, *p* < 0.001), and from the pigs fed the control mixture (0.007 mg/kg, *p* < 0.001). The mercury concentration in the muscles of pigs fed the hybrid rye mixture was under the limit of quantification (Table 6). The concentrations of cooper in diaphragmatic muscles of pigs fed the control and population rye mixtures (13.7 and 15.6 mg/kg, respectively) were the highest and differed significantly from lower values found in pig fed the hybrid rye mixture (8.09 mg/kg, *p* < 0.01). The concentration of zinc and manganese in the diaphragmatic muscle oscillated from 128.2 to 152.9 mg/kg and 1.06 to 1.19 mg/kg, respectively, and there were no significant differences between groups. The iron concentration in diaphragmatic muscles was the lowest in the pigs fed the control mixture (80.1 mg/kg) and in the pigs fed the population rye mixture (81.3 mg/kg) and differed from higher values found in the pigs fed the hybrid rye mixture (119.5 mg/kg, *p* < 0.05).

### 3.2. Concentration of Heavy Metals in Liver

The concentration of heavy metals in the liver of pigs fed a control and experimental mixtures did not differed significantly between pigs fed control and experimental mixtures, except for lead (Table 7). The highest Pb concentration was found in the liver of pig fed the control mixture (0.76 mg/kg) and it differed significantly from the lowest value found in the liver of pigs fed the population rye mixture (0.27 mg/kg, *p* < 0.001).

### 3.3. Concentration of Heavy Metals in Kidney

The concentration of cadmium in the kidneys was the highest in the pigs fed the population and hybrid rye mixtures (0.095 and 0.077 mg/kg, respectively) and differed significantly from the pigs fed the control mixture (0.038 mg/kg, *p* < 0.001) as shown in Table 8). On the other hand, the highest concentration of lead was found in the kidneys of pigs fed maize mixture and control mixture (1.59 and 0.60 mg/kg, respectively). This differed significantly from the lowest concentration found in the kidneys of pigs fed the hybrid rye (0.20 mg/kg, *p* < 0.001). Kidney copper concentration was the highest in the pigs fed the control mixture (35.8 mg/kg) and it differed significantly from the lowest value 23.5 mg/kg (*p* < 0.002) in the kidneys of pigs fed the population rye mixture. The highest concentration of zinc was found in the kidneys of the pigs fed the hybrid rye and population rye mixtures (130.6 and 127.8 mg/kg, respectively) and these values differed significantly from the lowest concentration found in the kidneys of pigs fed the maize mixture (107.5 mg/kg, *p* < 0.01; Table 8). The concentration of iron did not differed significantly between the pigs fed control and experimental feed mixtures. The highest concentration of Mn was found in the kidneys of pigs fed the population rye mixture (8.80 mg/kg). However intermediate Mn concentration was found in the kidneys of pigs fed hybrid rye (7.56 mg/kg, *p* < 0.001) and maize (6.74 mg/kg, *p* < 0.001), the lowest value was found in the pigs fed the control mixture (6.13 mg/kg, *p* < 0.001; Table 8).

### 3.4. Correlations of Heavy Metals Content in the Tissues

The Spearman’s correlation analysis of was performed between the concentration of heavy metals in the diaphragmatic muscle, liver and kidneys of pigs fed control and experimental feed mixtures (Table 9). Negative correlation was found between Cd concentration in kidneys and muscles of pigs fed hybrid rye (−0.820) and between Cu concentration in kidneys and muscles of pigs fed the control mixture (−0.786). Positive correlations were found between Zn concentration in liver and kidneys of pigs fed the control mixture (0.881) and the pigs fed the hybrid rye mixture (0.762). A positive correlation was found between Fe concentration in liver and kidneys of pigs fed the hybrid rye mixture (0.786).

## 4. Discussion

One of major challenges for human and animal health is environmental contamination with heavy metals. It is dangerous because they enter the food chain and can accumulate in plants and animal tissues leading to increased health risk for humans [27,28]. The content of heavy metals in food products from an animal’s origin depends on their level in the feed. This mainly depends on the ability of the crop plants to incorporate the elements into their tissues and the amounts of available heavy metals in the soil [9,29].

The concentration of examined heavy metals in the grains of cereals and soya bean meal used for the confectioning of feed mixtures used in the study was of varied quantity but they did not exceed the permissible levels according to European Commission Regulation (EC) No 1881/2006 [21]. However the levels of cadmium and lead in the grains of population and hybrid rye varieties and soya bean meal used in the studies were much lower than that in barley, wheat and maize. This might be in part due to peculiar properties of these plants to incorporate and accumulate less amounts of undesired heavy metals. The studies of Wieczorek et al. [30] reported similar levels of Cd and Pb in wheat and barley grains grown in the northeastern part of Poland. Mercury was not detected in the grains used for feed mixtures used in the study. The level of heavy metal in cereal grains used worldwide for feed production varies in different parts of the world [31,32]. An investigation of cadmium and lead uptake into wheat and barley performed in Great Britain between 1998 and 2000 reported that, in general, wheat had higher grain concentrations of cadmium than barley, and both species had low concentrations of lead, which were below the European Commission Regulation specifying the maximum permissible contaminant levels in foodstuffs [33].

The level of essential heavy metals in all grains and soya bean meal was under the range of allowance of the European community and varied in the different species examined. Soya bean meal showed highest amounts of these elements confirming the usefulness of this product in animal nutrition. On the other hand, the levels of essential metals in new rye varieties used in the study were comparable to levels in other cereal grains indicating that they are a good nutrient source for feed mixtures.

Heavy metals concentrations in the feed mixtures used in the study did not exceed the permissible levels according to the European community rules. Among the different feed mixtures used in the study the feed mixture with a maize inclusion of 60% of the ratio was characterized by higher levels of cadmium in comparison to control feed and rye varieties at the same level of inclusion. This observation provides important support for the beneficial use of new rye varieties able to accumulate less cadmium in their grains and, therefore, safer as a source of nutrients in pig grower and finisher feeding mixtures.

All concentrations of heavy metals found in the muscles, liver and kidneys of pigs fed control and experimental feed mixtures were in the range of allowable levels according to European community rules. The levels of cadmium and lead corroborate with data found by Phillips et al. [34], Leontopoulos et al. [35], and Pei et al. [36]. The levels of essential metals (Fe, Zn, Cu) were in the range of physiological values and indicate that feed mixtures containing a 60% inclusion of new rye varieties fulfil the nutritional demands of pigs during the fattening period [26].

Several studies show a linear relationship between dietary cadmium intake in livestock and Cd accumulation in organs like the liver and kidney. It seems to be directly related to the level and duration of exposure [37,38,39,40]. However, the concentration of cadmium in the muscles is lower and seems to be influenced by the rate of absorption, metallothioneins (MT) and iron status in the body [38,41]. In general, this is in line with the observations in our study, which showed the highest concentration of cadmium in the kidney, lower in the liver, and the lowest in the muscles. There were differences between the concentration of Cd in the kidneys of pigs from different feeding treatments being the highest in the ones fed population rye. However, Cd concentration in the liver of pigs fed the control mixture was 13% greater than that in the kidney. On the other hand, Cd concentration in the liver of pigs fed feed mixtures containing maize, population rye and hybrid rye was lower than in the kidney (63%, 60% and 47%, respectively). The muscles accumulated less Cd in comparison to kidneys and it was lower at the range of 92.1%, 90%, 77.9% and 89.6% for pigs of the control, maize, population rye and hybrid rye. Despite the highest Cd concentration being found in the kidneys and muscles of pigs fed population rye feed mixture, the percentage of Cd accumulated in muscles was similar to that observed in other groups. It could suggest an efficient Cd elimination by kidneys in pigs fed rye as well.

Biehl and Buck [42] stated that animal tissues with the highest concentrations are liver, kidney and bone. On the other hand, Philips et al. [34] reported increased levels of lead and cadmium in pig tissues fed mixtures enriched with these metals and indicated that the most sensitive tissues for cadmium and lead accumulation were the kidney, liver, hair and teeth; however, Pb accumulation in the kidney was much faster than in the liver.

Lead concentration found in the kidney liver and muscles of pigs in our study was generally in line with this statement. However, pigs fed the mixture containing population rye accumulated almost the same amount in the kidney (6% less) and in the liver. By contrast with this, pigs fed mixtures containing hybrid rye accumulated 50.5% less Pb in the kidney than in the liver. Control pigs were able to accumulate 21% less Pb. In the pigs fed 60% maize in the mixture a 2.7-fold Pb higher concentration was found in the kidney than in the liver. Stavreva-Veselinovska and Zivanovic [43] reported double the concentration in the kidney compared to that in the liver of pigs.

Mercury concertation in examined grains and feeds was under the limit of detection. This is because the most common source of mercury in feed materials is fishmeal [44,45], which was not included in experimental feeds. Moreover, Hang et al. [46] showed that there is no clear association with Hg between crops and soil, indicating that mercury in crop grains is mostly affected by other factors besides soil mercury. Therefore, when there is no external Hg contamination of the feed, elevated levels of mercury are not observed in tissues of domestic livestock under practical conditions [44,47].

In the present study some correlations were observed between the concentrations of metals in the studied organs. A greater number of correlations regarding Cd, Zn and Fe was found in the organs of pig fed the mixture containing 60% of hybrid rye. This could indicate there is a tendency to create a pattern of metals accumulation in these organs, which can be modulated by bioactive compounds present in hybrid rye grains.

Considering the presence of heavy metals in the environment and the diverse anthropogenic activities such the usage of pesticides containing undesired amounts of heavy metals, it is difficult to eliminate the entrance of these elements into the food chain. Exceeding the contamination limits may not be a problem if animals do not show signs of poisoning. However, this is rather shortsighted since animals have relatively short lifespans whereas the accumulation of undesired heavy metal can lead to disease states in the long term with the toxic effects appearing not in animals, but in humans consuming products of animal origin. Rye varieties that are cultivated on poorest soils, using the lowest amount of agrochemicals and that use a relatively low amount of water, may contribute to reducing heavy metals entering the food chain.

## 5. Conclusions

The present study shows that feeding mixtures containing 60% of hybrid rye grains resulted in reduced levels of lead in the muscles, liver and kidneys in comparison to control feed containing soya bean meal and barley, and maize, which are known to accumulate more heavy metals than other cereals. Regarding cadmium levels in the examined organs there is no clear effect on the concentration of this element in the muscles and in the liver depending on the type of feed mixture offered. Despite this, higher Cd concentration in the kidneys of pigs fed mixtures containing 60% of rye and maize may reflect the intensity of the renal elimination of this element. Moreover, as rye grains contain abundant amounts of essential metals needed for proper functioning of the whole body, new rye varieties, especially of hybrid type, could be used as an alternative source of cereal grains for pig nutrition.

## Figures and Tables

**Table 1:** Composition of the grower mixtures used in control and experimental groups of pigs during the first 40 days of fattening.

Item	Grower Diets			
	Control	Maize	Population Rye	Hybrid Rye
Ingredient (%)				
Soybean meal	18.50	20.80	20.50	20.50
Barley	38.00	6.00	6.40	6.40
Wheat	38.00	6.00	6.40	6.40
Rye	-	-	60.00	60.00
Maize	-	60.00	-	-
Wheat bran	1.10	3.8	2.00	2.00
Oil	1.20	0.20	1.50	1.50
Acidiffer	0.20	0.20	0.20	0.20
Universal complementary feed	3.00	3.00	3.00	3.00
Nutrient composition				
Metabolic Energy (MJ)	13.02	13.12	13.09	13.11
Dry matter (%)	87.52	87.41	87.59	87.59
Ash (%)	5.52	5.13	5.46	5.45
Protein (%)	17.56	17.54	17.37	17.40
Fat (%)	2.96	3.24	3.08	3.06
Fiber (%)	3.93	2.97	2.85	2.88
Starch (%)	41.14	44.58	38.97	39.13
Sugar (%)	3.73	3.42	5.59	5.75

**Table 2:** Composition of the grower mixtures used in control and experimental groups of pigs during the first 40 days of fattening.

Item	Finisher Diets			
	Control	Maize	Population Rye	Hybrid Rye
Ingredient (%)				
Soybean meal	9.40	12.00	12.00	12.00
Barley	41.40	8.60	9.50	9.50
Wheat	41.40	8.60	9.50	9.50
Rye	-	-	60.00	60.00
Maize	-	60.00	-	-
Wheat bran	4.00	8.00	5.00	5.00
Oil	1.2	0.20	1.40	1.40
Acidiffer	0.10	0.10	0.10	0.10
Universal complementary feed	2.50	2.50	2.50	2.50
Nutrient composition				
Metabolic Energy (MJ)	12.97	12.99	13.01	13.03
Dry matter (%)	87.48	87.39	87.49	87.48
Ash (%)	4.80	4.49	5.73	4.71
Protein (%)	14.52	14.46	14.54	14.47
Fat (%)	3.06	3.50	2.94	2.96
Fiber (%)	4.24	3.46	3.25	3.22
Starch (%)	45.08	47.64	42.68	42.74
Sugar (%)	3.26	3.00	5.26	5.57

**Table 3:** The mean content of heavy metals (mg/kg) in grains, on a dry matter basis, used for preparing control and experimental feed mixtures.

Element	Source Material for Feed Mixtures					
	Barley	Wheat	Maize	Population Rye	Hybrid Rye	Soybean Meal
Cd	0.042	0.059	0.023	0.005	0.005	0.085
Pb	0.165	0.192	0.226	0.091	0.054	0.086
Hg	UDL	UDL	UDL	0.002	0.001	UDL
Cu	4.72	4.05	2.02	3.84	2.41	16.0
Zn	46.3	36.6	29.9	28.1	24.8	50.9
Fe	55.7	45.5	28.5	28.8	22.8	82.7
Mn	15.3	44.9	6.47	28.7	22.5	31.1

Data are means of three independent determinations. UDL—Undetectable level.

**Table 4:** The mean content of heavy metals (mg/kg) in grower mixtures, on a dry matter basis (b. wt.), used in control and experimental feeding of pigs.

Element	Feed Mixture			
	Control	Maize	Population Rye	Hybrid Rye
Cd	0.064	0.235	0.021	0.024
Pb	0.198	0.758	0.122	0.304
Hg	UDL	UDL	UDL	UDL
Cu	23.9	24.6	17.0	13.0
Zn	128.6	161.4	109.5	106.5
Fe	193.9	174.2	102.2	84.4
Mn	99.6	74.2	48.5	66.4

Data are means of three independent determinations. Control: barley and wheat 1:1; Maize: barley, wheat and maize 1:1:3; Population rye: barley, wheat and population rye 1:1:3; Hybrid rye: barley, wheat and winter hybrid rye 1:1:3. UDL—Undetectable level.

**Table 5:** The mean content of heavy metals (mg/kg) in finisher mixtures, on a dry matter basis (b. wt.), used in control and experimental feeding of pigs.

Element	Feed Mixtures			
	Control	Maize	Population Rye	Hybrid Rye
Cd	0.125	0.137	0.009	0.045
Pb	0.147	0.666	0.125	1.235
Hg	UDL	UDL	UDL	UDL
Cu	27.27	35.79	11.23	13.65
Zn	179.4	155.4	80.1	89.1
Fe	206.57	202.97	85.93	97.53
Mn	93.93	113.70	58.82	72.00

Data are means of three independent determinations. Control: barley and wheat 1:1; Maize: barley, wheat and maize 1:1:3; Population rye: barley, wheat and population rye 1:1:3; Hybrid rye: barley, wheat and winter hybrid rye 1:1:3. UDL—Undetectable level.

**Table 6:** The mean content of heavy metals (mg/kg dry tissue, b. wt.) in the muscles of pigs fed control and experimental diets.

Element	Feed Mixture				SEM	*p*-Value
	Control	Maize	Population Rye	Hybrid Rye		
Cd	0.0029 ^a^	0.0056 ^a^	0.0212 ^b^	0.0076 ^a^	0.0031	0.001
Pb	0.0858 ^a^	0.3694 ^b^	0.2729 ^b^	0.4223 ^b^	0.0932	0.006
Hg	0.007 ^a^	0.032 ^b^	0.004 ^a^	UDL	0.0126	<0.001
Cu	13.7 ^b^	12.2 ^a,b^	15.6 ^b^	8.0900 ^a^	1.2538	0.002
Zn	128.2	139.0	152.9	152.1	7.6897	0.095
Fe	80.1 ^a^	93.7 ^a,b^	81.3 ^a^	119.5 ^b^	7.6581	0.004
Mn	1.11	1.19	1.35	1.06	1.1592	0.564

The data are shown as lsmeans (*n* = 8 for each group). Control: barley and wheat 1:1; Maize: barley, wheat and maize 1:1:3; Population rye: barley, wheat and population rye 1:1:3; Hybrid rye: barley, wheat and winter hybrid rye 1:1:3. UDL—Undetectable level. SEM—Standard error of means. ^a,b^ means with different superscripts within a row are significantly different (one-way analysis of variance (ANOVA) followed by a Tukey’s post hoc test (normally distributed data) or Kruskal–Wallis ANOVA with a Dunn’s post hoc test (for pairwise comparisons with at least one non-normally distributed dataset).

**Table 7:** The mean content of heavy metals (mg/kg dry tissue, b. wt.) in the liver of pigs fed control and experimental diets.

Element	Feed Mixture				SEM	*p*-Value
	Control	Maize	Population Rye	Hybrid Rye		
Cd	0.043	0.022	0.038	0.041	0.0107	0.715
Pb	0.756 ^c^	0.585 ^b,c^	0.273 ^a^	0.409 ^a,b^	0.0704	<0.001
Hg	UDL	0.002	0.001	UDL	0.0006	0.500
Cu	30.5	30.7	31.6	29.0	3.1893	0.950
Zn	229.8	208.1	206.7	221.9	19.278	0.799
Fe	485.7	593.5	604.6	461.8	90.483	0.588
Mn	11.3	11.0	9.08	10.1	0.5805	0.053

The data are shown as means (n = 8 for each group). Control: barley and wheat 1:1; Maize: barley, wheat and maize 1:1:3; Population rye: barley, wheat and population rye 1:1:3; Hybrid rye: barley, wheat and winter hybrid rye 1:1:3. UDL—Undetectable level. SEM—Standard error of means. ^a,b,c^ means with different superscripts within a row are significantly different (one-way ANOVA followed by a Tukey’s post hoc test (normally distributed data) or Kruskal–Wallis ANOVA with a Dunn’s post hoc test (for pairwise comparisons with at least one non-normally distributed dataset).

**Table 8:** The mean content of heavy metals (mg/kg dry tissue, b. wt.) in the kidneys of pigs fed control and experimental diets.

Element	Feed Mixtures				SEM	*p*-Value
	Control	Maize	Population Rye	Hybrid Rye		
Cd	0.038 ^a^	0.060 ^b^	0.095 ^b,c^	0.077 ^b^	0.0048	<0.001
Pb	0.596 ^b,c^	1.591 ^c^	0.254 ^b,a^	0.203 ^a^	0.1516	<0.001
Hg	0.002	0.002	UDL	UDL	0.0003	0.838
Cu	35.8 ^c^	34.0 ^b,c^	23.5 ^a^	24.6 ^a,b^	2.5498	0.002
Zn	116.1 ^a,b^	107.5 ^a^	127.8 ^b^	130.6 ^b^	5.0043	0.010
Fe	184.6	229.5	235.5	214.0	19.171	0.262
Mn	6.13 ^a^	6.74 ^a,b^	8.80 ^c^	7.56 ^b^	0.3093	<0.001

The data are shown as lsmeans (n = 8 for each group). Control: barley and wheat 1:1; Maize: barley, wheat and maize 1:1:3; Population rye: barley, wheat and population rye 1:1:3; Hybrid rye: barley, wheat and winter hybrid rye 1:1:3. UDL—Undetectable level. SEM—Standard error of means. ^a,b,c^ means with different superscripts within a row are significantly different (one-way ANOVA followed by a Tukey’s post hoc test (normally distributed data) or Kruskal–Wallis ANOVA with a Dunn’s post hoc test (for pairwise comparisons with at least one non-normally distributed dataset).

**Table 9:** The Spearman’s correlation coefficients of heavy metals in the muscles, liver and kidneys of pigs fed control and experimental diets.

		Group							
Element		Control		Maize		Population Rye		Hybrid Rye	
	tissue	muscle	liver	muscle	liver	muscle	liver	muscle	liver
Cd	liver	0.275		−0.188		0.283		0.060	
	kidney	0.242	−0.180	−0.217	0.108	0.323	−0.300	−0.820 *	0.239
Pb	liver	−0.667		0.071		−0.119		0.071	
	kidney	−0.097	−0.024	−0.524	−0.310	−0.310	−0.667	0.239	0.048
Hg	liver	0.200		ND		0.500		ND	
	kidney	ND	ND	ND	ND	−0.107	−0.771	ND	ND
Cu	liver	−0.286		−0.214		−0.143		0.452	
	kidney	−0.548	0.667	−0.786 *	0.429	−0.333	−0.524	−0.048	0.167
Zn	liver	0.214		0.143		0.548		−0.215	
	kidney	−0.119	0.881 *	0.146	−0.293	0.286	0.333	0.024	0.762 *
Fe	liver	0.095		−0.238		−0.524		0.381	
	kidney	−0.095	0.214	0.024	−0.119	−0.143	0.191	0.429	0.786 *
Mn	liver	0.214		0.167		0.690		0.119	
	kidney	0.000	−0.084	−0.503	0.227	0.239	0.381	0.071	−0.405

Control: barley and wheat 1:1; Maize: barley, wheat and maize 1:1:3; Population rye: barley, wheat and population rye 1:1:3; Hybrid rye: barley, wheat and winter hybrid rye 1:1:3. ND—not done. *—correlation statistically significant (*p* < 0.05).

## Data Availability

The data presented in this study are available on request from the corresponding author.
